# The Role of Foxes in Transmitting Zoonotic Bacteria to Humans: A Scoping Review

**DOI:** 10.1111/zph.13230

**Published:** 2025-06-13

**Authors:** Emma C. Hobbs, Bridgette McNamara, Sarah Hayman, Kim Blasdell, Eugene Athan, Daniel P. O'Brien, Michael Muleme

**Affiliations:** ^1^ Melbourne Veterinary School University of Melbourne Werribee Victoria Australia; ^2^ Centre for Innovation in Infectious Disease and Immunology Research (CIIDIR), School of Medicine, Deakin University Geelong Victoria Australia; ^3^ Barwon South West Public Health Unit Barwon Health Geelong Victoria Australia; ^4^ Library Service Barwon Health Geelong Victoria Australia; ^5^ Australian Centre for Disease Preparedness Commonwealth Scientific and Industrial Research Organisation (CSIRO) Victoria Australia

**Keywords:** bacterial zoonoses, foxes, humans, infectious disease transmission, scoping review

## Abstract

Zoonotic diseases inflict substantial burdens on human and animal populations worldwide, and many of these infections are bacterial. An Australian study investigating environmental risk factors for Buruli ulcer in humans detected the causative agent, *Mycobacterium ulcerans* , in the faeces of wild foxes, a novel finding that suggests foxes may be implicated in the transmission of this zoonotic bacterium. The aim of this scoping review was to systematically search and examine the global data for reports implicating foxes in the transmission of zoonotic bacteria to humans. A pre‐tested search strategy was implemented in five bibliographic databases (PubMed, Embase, CAB Abstracts, Cochrane Trials, Google Scholar). Eligible studies presented primary research data about zoonotic bacterial diseases that were confirmed or presumed to have been transmitted via foxes (excluding exclusively blood‐ or vector‐borne bacteria), with no restrictions on geographical setting or publication year. The final dataset included ten primary research articles, with varying study designs, settings, populations and testing methods. The described bacterial zoonoses were anthrax, cutaneous diphtheria, leptospirosis, faecal coliforms including *E. coli* , tularaemia, yersiniosis, and Buruli ulcer (the study that was the impetus for this scoping review). Fox‐human bacterial transmission was confirmed in one human case and considered likely to have occurred in certain high‐risk groups in another. The likelihood of fox‐human transmission having occurred in the remaining studies was possible (*n* = 5) or unlikely (*n* = 3). Identified and hypothesised drivers of fox‐human transmission included accidental and occupational factors. Published reports of fox‐human transmission of zoonotic bacteria are few, and generally indicative of relatively low risk. However, foxes can transmit zoonotic pathogens including bacteria to humans in a variety of settings, and human‐fox encounters are likely to increase with ongoing anthropogenic activities. Further research and public education campaigns would help increase knowledge and awareness of fox‐associated zoonoses.

**Summary**

- This scoping review examined the global literature describing confirmed or suspected transmission of zoonotic bacteria from foxes to humans.
- The small total number of published articles describing fox‐associated transmission or exposure to zoonotic bacteria in humans (*n* = 10) highlights a gap in knowledge about the role of foxes in transmitting bacterial zoonoses to humans.
- Foxes are widespread throughout human habitats and do pose a risk of transmitting zoonotic bacteria (and other infectious agents) to humans.
- People in high‐risk settings such as certain occupations or geographic locations should consider implementing protective measures to reduce zoonotic disease risk.

## 1 Introduction

It is an often‐cited statistic that approximately 60% of emerging infectious diseases are zoonotic (transmissible from animals to humans), and that of those, over 70% had wild animal origins (Allen et al. 2017; Jones et al. 2008; Karesh et al. 2012; Wolfe et al. 2007). Zoonotic agents encompass the full range of pathogenic organisms, including bacteria, viruses, parasites, protozoa, fungi, and prions, and can be transmitted by various routes, including direct, indirect, and foodborne pathways. Globally, zoonotic diseases cause considerable morbidity and mortality in both human and animal populations, impose substantial economic burdens on public health and agricultural sectors, and can affect tourism and trade at multiple levels (Christou 2011; Rahman et al. 2020). One study estimated the burden of 56 zoonotic pathogens at 2.5 billion human infections and 2.7 million human deaths a year (Grace et al. 2012). The recent COVID‐19 pandemic, the origins of which were epidemiologically traced to a live wild animal market in the Chinese city of Wuhan (Crits‐Christoph et al. 2024), emphasised the potentially disastrous and far‐reaching consequences of zoonotic disease spillover from wildlife. The (re‐)emergence and spread of wildlife‐origin zoonoses have many known anthropogenic drivers, including increasing human population density, deforestation and urbanisation, increased global trade and travel, illegal trade and consumption of wildlife, climate change, and intensive agricultural practices, particularly in tropical regions with high wildlife biodiversity (Horby et al. 2014; Karesh et al. 2012; Taylor et al. 2001; Wolfe et al. 2007). Of the known zoonotic pathogens, bacteria reportedly account for between one‐third (Taylor et al. 2001) to just over half (Jones et al. 2008). Bacterial zoonoses of major public health significance include anthrax (*Bacillus anthracis*), brucellosis (*Brucella abortus*, *B. melitensis* , *B*. *suis* , *B*. *canis* ), Q fever (*Coxiella burnetii*), leptospirosis (*Leptospira* spp.), plague (*Yersinia pestis*), *Staphylococcus aureus* including the methicillin‐ and multi‐drug resistant strains (MRSA, MDRSA), and mycobacterial diseases including zoonotic tuberculosis (*Mycobacterium bovis*, *M. caprae* , *M*. *microta*) and zoonotic leprosy (*M. leprae*, *M*. *lepromatosis*) (Christou 2011; Meyers et al. 1991; Neyra et al. 2014; Rahman et al. 2020; Schilling et al. 2019; Sharma et al. 2015).

Foxes are one of the most widely distributed mammals, found on every continent except Antarctica. There are twelve ‘true foxes’ of the *Vulpes* genus, and members of the *Lycalopex*, *Cerdocyon*, *Otocyon*, and *Urocyon* genera are sometimes called ‘false foxes’. Distribution and abundance vary greatly by species. The arctic fox (*Vulpes lagopus*) is restricted to the circumpolar Arctic tundra regions of North America, Eurasia and Fennoscandia (IUCN 2024), while the diminutive Cozumel fox (*Urocyon cinereoargenteus*), considered critically endangered if not extinct for the past two decades, is only found on one small Mexican island (Cuarón et al. 2004). The most widely distributed species is the red fox (*Vulpes vulpes*), covering an estimated 70 million km^2^ across most of the northern hemisphere (IUCN 2024), and established across Australia following deliberate release by British settlers in the mid‐1800s for sport and to control introduced rabbit (*Oryctolagus cuniculus*) populations (Kamler and Ballard 2002; Saunders et al. 2010). Foxes are flexible and opportunistic predators, known to consume a wide variety of small and large mammals, birds and invertebrates, as well as carrion, fruits and berries (Castañeda et al. 2022; Fleming et al. 2022; Triggs et al. 1984). Red foxes have been responsible for the decline or extinction of some small canids and ground‐nesting birds in North America, and several native species of birds, reptiles and mammals in Australia (Dickman 1996; Kamler and Ballard 2002; Saunders et al. 2010). Highly adaptable to a range of environments (IUCN 2024), foxes are increasingly reported in urban and agricultural areas where anthropogenic food sources and refugia can allow them to attain population densities far higher than in rural areas (Bateman and Fleming 2012; Marks and Bloomfield 1999; Scott et al. 2014).

The wide geographical distribution of foxes and their proximity to people poses a risk of zoonotic disease transmission via both direct (e.g., bites and scratches, contact with infected skin, tissues or fluids) and indirect methods (e.g., contamination of the environment, or by intermediate vectors). Well‐documented examples of fox‐transmissible zoonoses include rabies (Christie 1981; Fooks et al. 2017; Nurumal et al. 2022), visceral leishmaniasis (Karayiannis et al. 2015; Mohebali et al. 2018), infections with *Cryptosporidium* and *Giardia*, and with numerous species of nematode, cestode and trematode, some of which can be fatal (Conraths et al. 2017; Craig 2003; Holland et al. 2024; Karamon et al. 2018; Oksanen et al. 2016; Perera et al. 2024; Veronesi et al. 2023). Red foxes are also susceptible to SARS‐CoV‐2 and the highly pathogenic H5N1 avian influenza virus (Bordes et al. 2023; Porter et al. 2022).

Buruli ulcer (BU) is a necrotising bacterial skin disease caused by *Mycobacterium ulcerans* . Reported in over 30 countries (WHO 2023), it is only recognised as a zoonosis in Australia, where human cases are preceded by the emergence of *M. ulcerans* in native possum populations (McNamara et al. 2025). Phylogeographic and phylogenetic analyses indicate that *M. ulcerans* is most likely a point source pathogen, introduced to new areas and subsequently spreading, rather than a ubiquitous pathogen that lies dormant until triggered by environmental disturbances or other factors (Buultjens et al. 2018). Recent research, including some conducted by our group, has confirmed the role of mosquitoes and native possums in the transmission of *M. ulcerans* in southeast Australia (Fyfe et al. 2010; Johnson et al. 2007; Lavender et al. 2011; Mee et al. 2024; O'Brien et al. 2014). However, the mechanisms by which *M. ulcerans* is introduced into new geographic areas are not adequately explained at present. Mosquitoes can function as mechanical vectors for the bacteria, but the low pathogen load they carry (Mee et al. 2024) is unlikely to be sufficient to seed new outbreaks. Possums are known to excrete significant numbers of *M. ulcerans* into the environment (Blasdell et al. 2022; Fyfe et al. 2010; Hobbs et al. 2024) and are likely contributing to the zoonotic spillover of BU to humans in endemic areas, but are highly territorial and tend to remain within small home ranges (typically spanning one to two hectares in urban areas (Harper 2005)). Foxes are known to predate on possums (Dickman 1996; Triggs et al. 1984) and also roam large distances, with reported home ranges from 100 to over 10,000 ha (Carter et al. 2012; Kobryn et al. 2023; Lindsø et al. 2022), making them potentially capable of introducing novel pathogens into new geographical areas via direct and indirect pathways, including faecal environmental contamination. The novel finding from our group that 30% of fox faeces (6/20) were positive for *M. ulcerans* DNA, with evidence of bacterial viability on further testing (Blasdell et al. 2022) suggests foxes may be implicated in the environmental transmission of *M. ulcerans* in southeastern Australia and potentially in other BU endemic parts of the world.

An informal literature search revealed no reports of fox involvement in *M. ulcerans* transmission in other BU endemic areas, and that in general, reviews describing the role of foxes in the transmission of bacterial zoonoses are lacking. The aim of this scoping review was to systematically examine and summarise the existing global literature on the role of foxes in the transmission of zoonotic bacterial diseases to humans, to better understand shedding patterns and transmission pathways that may be applicable to BU.

## 2 Materials and Methods

### 2.1 Review Protocol, Team and Expertise

The review team included individuals with multidisciplinary expertise in epidemiology, microbiology, veterinary medicine, knowledge synthesis, and information science. The scoping review protocol was developed by the review team using the Preferred Reporting Items for Systematic reviews and Meta‐Analysis extension for Scoping Reviews (PRISMA‐ScR) guidelines and checklist ((Tricco et al. 2018), Appendix S1), and pre‐tested prior to implementation to ensure reproducibility, transparency, and consistency of the review methods. The final protocol was registered with the Open Science Framework on 27th December 2024 (https://osf.io/ezp52/).

### 2.2 Review Question and Scope

The following research question was formulated using the PCC framework (Peters et al. 2020): by what mechanisms have foxes been implicated in transmitting zoonotic bacteria, such as *M. ulcerans* , to humans?

The PCC elements of our scoping review were as follows:

1. Population/problem = humans and foxes (*Vulpes, Cerdocyon, Otocyon, Lycalopex* and *Urocyon* spp.)
2. Concept = exposure or transmission of zoonotic bacteria (excluding exclusively blood‐ and vector‐borne bacteria) from foxes to humans
3. Context = all regions

### 2.3 Search Strategy

The search strategy was devised by the review team and pre‐tested in PubMed and Embase to refine and determine the most effective balance of sensitivity and specificity in the identification of potentially relevant articles. As our review was focused on bacterial zoonoses that are transmitted by direct or environmental pathways, we excluded exclusively blood‐ or vector‐borne zoonotic bacteria due to the differences in transmission dynamics and the unique ecological factors involved in their spread. The final search strategy was implemented in five bibliographic databases: PubMed, Embase, Cochrane Trials and CAB Abstracts; and Google Scholar, which includes grey literature. The PubMed, Embase and Cochrane Trials searches were conducted on 5th June 2024 and the CAB Abstract and Google Scholar searches were conducted on 6th June 2024.

The search terms were the same for all databases, but the formatting of the terms varied due to different database architecture. When applicable, search terms included subject heading search, such as Medical Subject Headings (MeSH) from PubMed. The general search strategy used in this review was #1 AND #2 AND #3, where:

- #1: Exp foxes/or (fox or foxes or vulpes* or cerdocyon or lycalopex or otocyon or urocyon or dusicyon).mp.
- #2: Exp Bacterial zoonoses/or exp. Gram positive bacteria/or exp. Gram negative bacteria/or exp. Gram positive infections/or exp. Gram negative infections/or exp. Bacterial toxins/or (“bacterial zoonoses” or “gram positive and gram negative bacteria” or “gram positive bacteria” or “gram negative bacteria” or anthrax or anthrax* or anthracis or Bordetel* or botulism or brucell* or Burkholderi* or Buruli or campylobacter* or cholera or clostridium or corynebacteri* or coxiell* or cryptospor* or diphtheri* or “e.coli” or “ *escherichia coli* ” or erisypel* or escherichia or francisell* or glanders or helicobacter* or klebsiell* or leprosy or leptospir* or listeri* or melioid* or MRSA or “methicillin resistant staphyolococcus aureus” or mycobacteri* or paratuberculos* or pasteurell* or plague or pleuropneumonia or “q fever” or salmonell* or staphylococc* or streptococc* or tuberculo* or tularaemia or tularemia or weil* or yersini*).mp.
- #3: Exp transmission/or (transmission or spread or transmit* or reservoir* or epidemiolog* or host* or outbreak* or role* or sentinel or spillover or shed*).mp.

The search strategies and results are available in Appendix S2.

### 2.4 Eligibility Criteria and Relevance Screening

The eligibility criteria for our scoping review were:

1. Relevance to the PCC elements, requiring evidence of zoonotic transmission of or exposure to bacteria from foxes to humans (publications were excluded that: identified potentially zoonotic bacteria in foxes but did not also test samples from humans or from human environments; presented data about transmission of bacteria from humans to foxes (anthroponotic transmission); presented data about exclusively blood‐ or vector‐borne zoonotic bacteria; presented data about non‐bacterial zoonoses);
2. Publications in English, and for which full text was available; and
3. Articles reporting primary research data (review and opinion articles were excluded).

No restrictions were placed on publication date or geographical location of studies.

All articles retrieved from the electronic searches were imported into reference management software (EndNote, Clarivate Analytics, Pennsylvania, USA) and subjected to the automated de‐duplication process. The resultant files were uploaded into a web‐based electronic systematic review program (Covidence, Veritas Health Innovation, Melbourne, Australia), designed specifically for screening and data extraction of systematic and scoping reviews. Manual de‐duplication was conducted on the imported studies to obtain the final study set.

Article screening for relevance to the PCC criteria was conducted in two stages: title and abstract screening, in which articles were marked as ‘Yes’, ‘No’ or ‘Maybe’; and full‐text screening, during which full‐text articles were read and marked ‘Include’ or ‘Exclude’, with those marked for exclusion also assigned one of four reasons. Data screening instructions are presented in Appendix S3. For both stages, articles were screened by two independent reviewers to reduce the possibility of excluding relevant reports. One primary reviewer (ECH) conducted the first screening rounds, and the second rounds were shared by two secondary reviewers (MM and BM). After each screening stage, the three reviewers met to discuss and resolve disagreements on article status. The studies deemed to have met all eligibility criteria were then subjected to data extraction.

### 2.5 Data Extraction and Analysis

The data extraction form was developed by the review team, and was pre‐tested and refined over several iterations prior to use. The final version (Appendix S4) included sections for study design and methodology, locations (geographical, categorical e.g., urban, rural) and populations (humans, foxes, other animals); bacterial zoonotic disease(s) described in the study; clinical presentation in host and reservoir species; details of sample testing and results, including routes of infection, shedding and exposure; risk factors for transmission, if known; and an assessment of the strength of evidence for fox‐human zoonotic bacterial transmission. This latter assessment was categorised as: high for definite cases of zoonotic fox‐human transmission; medium where specific risk factors were identified; and low where transmission was suggested but no specific supporting data were provided. The low strength category was further subdivided into ‘possible’ or ‘unlikely’ to provide more nuanced differentiation, based on the conclusions drawn by the studies' authors.

The primary reviewer (ECH) extracted data from the final set of eligible full‐text articles into the data extraction template in Covidence using an iterative process as previously described (Peters et al. 2020). Any queries regarding data extraction were resolved by re‐checking the article in conjunction with the secondary reviewers. Given the limited resources and time available to the review team, the optional review step of critical appraisal of individual sources of evidence was not undertaken. Extracted data were exported from Covidence to an Excel spreadsheet (Microsoft Corporation, 2024). Data characterisation and descriptive analysis (frequencies and percentages) were conducted on the final dataset to allow categorisation and charting. Tables were generated to facilitate understanding of key trends and research outputs.

## 3 Results

### 3.1 Article Searches and Screening

**FIGURE 1:**
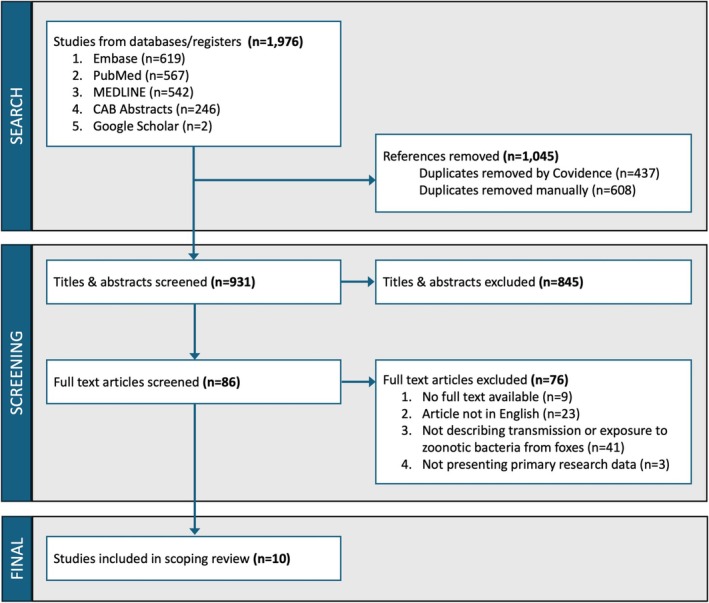
PRISMA diagram outlining the scoping review process.

A total of 1976 article citations were identified from the literature searches, of which 1045 were duplicates. Title and abstract screening was conducted on 931 studies, most (*n* = 845, 91%) of which were ineligible (largely due to reporting on non‐bacterial zoonoses, or in animals other than foxes) and were excluded. Full‐text screening examined 86 articles, and 76 were excluded, most commonly because they did not describe transmission or exposure of zoonotic bacteria from foxes (41/76, 54%). The final dataset comprised 10 studies that met our inclusion criteria (Figure 1) (Aikembayev et al. 2010; Blasdell et al. 2022; Ervin et al. 2014; Esmaeili et al. 2014; Gallagher et al. 2013; Moore et al. 2015; Silva et al. 2015; Silva et al. 2014; Tarek et al. 2023; Wojciech et al. 2004).

Calculation of inter‐rater reliability data (Cohen 1960) showed that concordance between reviewers during the title and abstract screening stage was, on average, fair, but was much improved (‘substantial’) during the full‐text screening round.

### 3.2 General Study Characteristics

Most reviewed studies (7/10, 70%) were published between 2010 and 2019, with two more recent studies and one published in the early 2000s (Table 1). Study locations varied, with three from Europe/UK (two of which were from London), two each from North and South America (both USA and Brazil) and Asia, and one from Australia. Studies were conducted in urban (*n* = 7), semi‐urban (*n* = 5) and rural (*n* = 8) settings, and one also included a beach setting. Four studies were set only in one location (two in cities, two in rural), whereas most spanned two or more settings, including one country‐wide study.

**TABLE 1:** Summary of general and specific study characteristics of the 10 studies examined in this scoping review.

General study characteristics	No.	%
Publication date		
2000–2009	1	10
2010–2019	7	70
2020–2024	2	20
Study location		
Europe & UK	3	30
North America	2	20
South America	2	20
Asia	2	20
Australia/New Zealand	1	10
Study setting^a^		
Urban	7	33
Semi‐urban	5	24
Rural	8	38
Other: beach	1	5
Study design^a^		
Environmental sampling	4	40
Cross‐sectional/prevalence study	4	40
Case report	2	20
Retrospective study	1	10
Pre‐post interventional comparison	1	10
Questionnaire survey	1	10

Specific study characteristics	No.	%
Zoonotic bacteria investigated		
*Leptospira*	3	30
Faecal indicator bacteria, incl. *E.coli*	2	20
*Anthrax*	1	10
*Mycobacterium ulcerans*	1	10
*Francisella tularensis*	1	10
*Corynebacterium ulcerans*	1	10
*Yersinia enterocolitica*	1	10
Fox species investigated^a^		
Red fox *(Vulpes vulpes)*	5	42
Crab‐eating fox *(Cerdocyon thous)*	2	17
Other/not stated	3	25
Arctic fox *(Vulpes lagopus)*	1	8
Grey fox *(Urocyon cinereoargenteus)*	1	8
Origin of foxes investigated		
Wild	8	80
Farmed	1	10
Unspecified	1	10
Origin of samples tested^a^		
Humans	7	32
Foxes	6	27
Other animals	6	27
Environment (water, soil, plants)	2	9
Insects	1	5
Types of samples tested^a^		
Blood	5	29
Water	3	18
Faeces	2	12
Urine	2	12
Soil	1	6
Plant material	1	6
Subcutaneous tissue	1	6
Tonsils	1	6
Insects	1	6
Sample testing methods used^a^		
Culture	6	32
PCR	5	26
Genotyping	3	16
ELISA	2	11
Microscopic agglutination test	2	11
MALDI‐TOF	1	5
Strength and likelihood of fox‐human transmission		
High, definite	1	10
Medium, likely	1	10
Low, possible	5	50
Low, unlikely	3	30
Risk factors for human exposure to zoonotic bacteria		
Direct		
Accidental (contact with/bite from infected fox)	3	30
Occupational (hunting, butchering, farming, eating meat of foxes)	2	20
Indirect		
Poor sanitation (contact with contaminated water)	4	40
Poor hygiene (contact with fox faeces)	1	10

Abbreviation: MALDI‐TOF, matrix‐assisted laser desorption/ionisation time‐of‐flight.

^a^ Multiple answers allowed per article (i.e., percentages exceed 100%).

Study designs were mixed, and three studies incorporated more than one method. Environmental sampling and cross‐sectional/prevalence studies were the most common, each being utilised in four studies. Two of the environmental sampling studies conducted microbial source tracking to identify sources of faecal contamination in drinking and recreational water sources. Two studies were case reports, both describing zoonotic bacterial infections in a (different) female patient in her mid‐60s who presented to a London hospital. Two of the cross‐sectional studies were set in Brazil and described random sampling of humans, domestic, and wild animals for the same disease but testing different sample types; one of these studies was a conference poster abstract and the other was a peer‐reviewed research article, both attributed to the same first author and sharing three other co‐authors.

### 3.3 Specific Study Characteristics

There were seven bacterial zoonoses described in the ten studies evaluated in this review. Leptospirosis was investigated in three studies, and faecal indicator bacteria, including *E. coli* in two, with the remaining five studies each involving a different zoonotic bacterium (Table 2). The complete data from the extracted studies is available in Appendix S5.

**TABLE 2:** Specific details of the 10 studies examined in this scoping review.

(Citation)	Country of study	Location and setting	Bacterial zoonosis (and agent) of interest	Samples tested	Human study population	Fox species	Fox origin	Nature of transmission or exposure from a fox(es)	Strength of association with a fox(es)	Likelihood of fox‐human transmission	Comments
(Aikembayev et al. 2010)	Kazakhstan	Country‐wide	Anthrax (*Bacillus anthracis*)	Archived *B. anthracis* cultures collected from people and animals	People (presumably residents of or visitors to Kazakhstan) from whom samples were collected	Red fox (*Vulpes vulpes*) Arctic fox (*Vulpes lagopus*)	Not specified	Direct contact with infected foxes or fox products	Low	Possible	This study mainly focused on mapping the geographical distribution of historical anthrax outbreaks in Kazakhstan. The number of foxes affected by or tested in the studied outbreaks was unclear
(Blasdell et al. 2022)	Australia	Urban coastal areas in Victoria	Buruli ulcer (*Mycobacterium ulcerans*)	Soil, water, mammalian faeces, plant material	(Residents of or visitors to the study area who had had Buruli ulcer (cases), and postcode‐matched negative controls)	Red fox (*Vulpes vulpes*)	Wild	Indirect contact with faeces from infected foxes	Low	Possible	Two of the authors of the current scoping review are co‐authors. The findings from this paper were the impetus for conducting this scoping review
(Ervin et al. 2014)	USA	Lower Arroyo Burro watershed, Santa Barbara County, central California	Faecal indicator bacteria (*Enterococci, E. coli*, total coliform bacteria)	Water	(Residents of or visitors to the study area)	Not stated	Wild	Indirect contact with water contaminated by faeces from infected foxes	Low	Possible	Pet dogs were the likely source of most canine faecal contamination, however the marker is also present in faeces of foxes, deer and racoon, so they may also be contributing
(Esmaeili et al. 2014)	Iran	Kurdistan province	Tularaemia (*Francisella tularensis*)	Human blood	Hunters & their families; butchers & slaughterhouse workers; health care workers; people referred to medical diagnostic laboratories	Not stated	Wild	Direct contact via hunting and/or eating meat of infected foxes	Medium	Likely	Study participants also filled out a questionnaire to identify demographic and occupational exposure factors
(Gallagher et al. 2013)	UK	London	Leptospirosis (*Leptospira icterohaemorrhagiae*)	Human blood and urine	66‐year‐old female who presented to hospital	Red fox (*Vulpes vulpes*)	Wild	Direct via bite from infected urban fox cub	High	Definite	Case report. Patient was bitten by a fox cub in her garden 2 weeks prior to onset of clinical signs
(Moore et al. 2015)	UK	London	Cutaneous diphtheria (*Corynebacterium ulcerans*)	Human blood and excised subcutaneous tissue	67‐year‐old female who presented to emergency department	Red fox (*Vulpes vulpes*)	Wild	Dierct or indirect contact with infected semi‐tame urban fox	Low	Possible	Case report. Patient was avid gardener and had extensive animal contact history but denied having received any bites or scratches
(F. Silva et al. 2015)	Brazil	Rio Grande do Sul state	Leptospirosis (*Leptospira interrogans*)	Blood from humans and animals (domestic and wild)	Rural farmers	Crab‐eating fox (*Cerdocyon thous*)	Wild	Indirect contact with urine and/or other excretions from infected foxes	Low	Unlikely	Authors concluded that results indicate nutria (*Myocastor coypus*) were the most likely bacterial reservoir in this setting
(F. J. Silva et al. 2014)	Brazil	Rural areas within nine states	Leptospirosis (*Leptospira* spp.)	Urine from humans and animals (domestic and wild)	Residents of or visitors to the study areas	Crab‐eating fox (*Cerdocyon thous*)	Wild	Indirect contact with urine and/or other excretions from infected foxes	Low	Possible	A total of 20 bacterial strains were identified, from 2 humans, 1 horse, 1 sheep, 1 cow, 1 dog, and an unspecified number of wild mammals
(Tarek et al. 2023)	USA	West Run watershed, West Virginia	*E. coli*	Water	(Residents of or visitors to the study area)	Red fox (*Vulpes vulpes*) Grey fox (*Urocyon cinereoargenteus*)	Wild	Indirect contact with water contaminated by urine and/or other excretions from infected foxes	Low	Possible	Authors concluded pet dogs were the likely source of most canine faecal contamination, but foxes may also be contributors
(Wojciech et al. 2004)	Poland	Lower Silesia	Yersiniosis (*Yersinia enterocolitica*)	Human faeces, fox faeces, pig tonsils	Hospital patients	Not specified	Farmed	Direct or indirect contact with infected foxes or fox products	Low	Unlikely	The data support the general view that pigs are the major reservoir of these bacteria for humans

#### 3.3.1 Study Populations

The human populations were mainly residents of (or presumably visitors to) the study areas during the study periods, with a specific subset in one environmental sampling study being the properties of people who had had the disease under investigation (cases) and their postcode‐matched controls. The number of humans that were sampled was quantified only in three studies: the two case studies that each described a single human case, and the Iranian tularaemia study that tested 250 humans who were differentiated into groups based on demographic and behavioural risk factors. An unspecified number of hospital patients made up the human testing cohort in the Polish study. Three of the other examined studies did not test humans, but rather tested environmental samples from human environments.

The country‐wide Kazakhstani study utilised 93 archived cultures and data from the Kazakhstan National *Bacillus anthracis* collection, stating that most isolates came from (an unspecified number of) human patients. Some isolates came from the blood or organs of ruminants, and “a few from soil or other inanimate objects contaminated by contact with blood or tissues of infected animals” (Aikembayev et al. 2010). A table entry stated 1 outbreak/sample in ‘fox’, in which there was 1 death/outbreak and 1 total death, and 2 outbreaks/samples in ‘arctic fox’, in which there were 5 deaths/outbreak and a total of 6 deaths; no further clarification was provided.

Of the fox species studied, half the studies involved the red fox (*Vulpes vulpes*), while the two Brazilian leptospirosis studies involved crab‐eating foxes (*Cerdocyon thous*). Arctic foxes (*Vulpes lagopus*) and grey foxes (*Urocyon cineroargenteus*) were each described in one study, both of which also involved red foxes. The fox species was unspecified in three studies. Eight studies definitively involved wild foxes; the Polish study described farmed foxes but did not specify the species, and the authors of the Kazakhstani study stated that “sporadic outbreaks occurred on mink farms and among foxes”, but the origin of the foxes was not specified.

#### 3.3.2 Sample Testing

The most tested analyte was blood/serum (*n* = 5, 29%), followed by water (18%), then faeces and urine (each 12%). Humans were the most frequently sampled host (*n* = 7, 32%), followed by foxes and other animals (each 27%). Many studies utilised more than one sample testing method, with bacterial culture the most used (*n* = 6, 32%), followed by PCR (26%).

#### 3.3.3 Prevalence and Significance of Infection in Study Populations

Besides the two case studies, which each tested samples from a single human patient, only two studies provided quantifiable data about the prevalence of infection or disease in the study populations. Blasdell et al. (2022) tested a variety of environmental samples at both case and control properties within the BU endemic areas studied. A variety of mammalian faeces were opportunistically collected for testing, and *M. ulcerans* was detected in 30% (6/20) of fox faecal samples. Esmaeili et al. (2014) tested a total of 250 human blood samples and determined that the seroprevalence of tularaemia in individuals exposed to foxes via hunting or eating the meat (25%) was significantly higher than in others (8.65%) (*p* = 0.01).

#### 3.3.4 Strength and Likelihood of Fox‐Human Bacterial Transmission

The strength of association between human zoonotic bacterial infection/disease and fox(es) was assessed to be low (likelihood either ‘possible’ or ‘unlikely’) in most (*n* = 8, 80%) of the studies, with one assessed as medium (‘likely’) and one as high (‘definite’). The ‘definite’ study was a case report that described leptospirosis (Weil's disease) in a 66‐year‐old woman who developed multisystemic symptoms two weeks after being bitten by a fox cub in her London garden (Gallagher et al. 2013). The Iranian study was assessed as ‘likely’ based on the stratification of humans that tested positive for antibodies specific for *Francisella tularensis* (tularaemia) into risk factor groups, which found the highest seropositivity in people who hunted, butchered and/or consumed the meat of foxes (Esmaeili et al. 2014).

Several studies presented data relating to environmental contamination (of soil, or sources of recreational or drinking water) by fox excreta, including faeces and urine. The two studies that utilised microbial source tracking to identify host sources of faecal contamination of waterways confirmed the presence of canine DNA markers in fox faeces in similar concentrations to those from domestic dogs, suggesting that foxes are possibly contributing to faecal contamination in those settings (Ervin et al. 2014; Tarek et al. 2023). However, the authors of both studies noted the limitations of using canine DNA markers to infer fox origin of faecal contamination: Ervin et al. (2014) also detected canine markers in faecal samples from coyote, deer and raccoon, while Tarek et al. (2023) discussed the occurrence of cross‐reactions with faecal samples from coyote, geese, humans, chickens and cows. The Kazakhstani study mainly focused on mapping the geographical distribution of historic anthrax outbreaks, and presented limited and at times unclear data relating to the involvement of foxes in the outbreaks. The Australian *M. ulcerans* paper investigated environmental risk factors in the residential properties of people previously confirmed with BU disease status and their postcode‐matched (negative) controls. A high proportion of fox faeces were PCR positive for *M. ulcerans* , suggesting that foxes are potentially contributing to the environmental contamination of this bacterium in this setting (Blasdell et al. 2022). These studies were assessed as being of low evidentiary strength, but fox‐human transmission was deemed possible.

The evidentiary strength of fox‐human transmission in the remaining three studies was also low, but deemed unlikely. The case study by Moore et al. (2015) described cutaneous diphtheria (*Corynebacterium ulcerans*) in a London woman in her mid‐60s, which was ascribed to a puncture wound visible on her hand. The injury was presumed to have come from an animal or environmental source, as the patient was an avid gardener and had had extensive contact with domestic and wild animals, including six pet dogs, sixteen pet or feral cats, and a semi‐tame fox that entered her house for food. The patient denied having been bitten or scratched by any of the animals, and veterinary testing of the contact animals was discussed but ultimately not attempted. Given the patient's extensive animal contact history, the probability that the fox was responsible for the zoonotic transmission in this case was assessed to be unlikely. The authors of the two remaining studies concluded that other animals (pigs, nutria (*Myocastor coypus*)) were more likely to have been the major bacterial reservoirs for zoonotic transmission to humans than foxes in those settings (F. J. Silva et al. 2014; Wojciech et al. 2004).

#### 3.3.5 Risk Factors Associated With Zoonotic Bacterial Infection From Foxes

Several potential risk factors were identified in the examined studies. Direct risk factors were contact with infected foxes or fox products via occupational (hunting, butchering, farming or eating meat of foxes) or accidental (e.g., bites or scratches from foxes while gardening) pathways. Increasing age was also found to be a risk factor in the Iranian tularaemia study (Esmaeili et al. 2014). Indirect risk factors included contact with fox faeces or urine via contaminated soil or water, attributable to poor sanitation or hygiene practices.

## 4 Discussion

In this scoping review, we systematically examined the global literature and identified ten primary studies implicating foxes in zoonotic transmission of bacteria to humans (excluding exclusively vector‐ or blood‐borne zoonotic bacteria). We found one definite case of zoonotic transmission of leptospirosis from a fox cub bite in London, and another study described likely zoonotic transmission of tularaemia in Iranian hunters, butchers, and consumers of fox meat. The remaining studies described possible or unlikely transmission of zoonotic bacteria from foxes to humans. These findings appear to be largely reassuring from a public health perspective, in that there are limited reports of fox‐human bacterial transmission.

The confirmed case of fox‐origin leptospirosis in London, however, provides a timely reminder that all wild animals can potentially harbour and transmit infectious pathogens, and that foxes are not restricted to semi‐rural or wild areas. There are significant resident red fox populations in many large cities across the UK, Europe, North America and Australia (Bateman and Fleming 2012; Dumas and Touma 2024; Marks and Bloomfield 1999; Scott et al. 2014), and ongoing urbanisation and encroachment of humans into wildlife habitats make human‐fox encounters increasingly likely. Urban fox densities typically range from 2 to 16 foxes per square kilometre (Heydon et al. 2000; Marks and Bloomfield 1999), but have been as high as 37 foxes per square kilometre in Bristol, UK (Baker et al. 2001).

While our scoping review did not identify reports of foxes involved in the transmission of *M. ulcerans* in other parts of the world where BU is endemic, this might reflect an absence of evidence rather than evidence of absence. In general, foxes—including native, nuisance, and farmed species—appear to be relatively poorly studied. In Australia, foxes likely ingest *M. ulcerans* via infected prey animals such as ringtail possums, a major component of their diet (Fleming et al. 2022; Triggs et al. 1984) and a known reservoir of *M. ulcerans* in Victorian BU endemic areas (Fyfe et al. 2010; Hobbs et al. 2024; O'Brien et al. 2014). At present, it is unclear whether foxes are merely passive carriers or play an active role in bacterial amplification and movement into new areas. Further research into the role of foxes in the transmission of zoonotic pathogens, including *M. ulcerans*, is needed.

Certain behaviours or risk factors may increase an individual's likelihood of acquiring zoonoses from foxes. Differing attitudes towards foxes may affect the frequency of human‐fox interactions, whether through attracting foxes to gardens and attempts at handfeeding or patting, or via aggressive confrontations with foxes in unwanted proximity to people, animals, or property. In Switzerland, 85% of surveyed households inadvertently provided food for foxes (e.g., via unsecured rubbish bins, compost heaps, and pet food (Contesse et al. 2004)), while two UK surveys reported that 10%–14% of British adults actively encouraged foxes to visit their gardens, typically by leaving out food, water, or toys (Baker et al. 2004; Guthrie 2019). Pets may also act as intermediaries, encountering zoonotic infections via contact with wild animals or contaminated environments and transmitting them to owners via direct and indirect pathways (Rahman et al. 2020).

Occupational zoonoses have long been recognised as health hazards for people who work with animals, including veterinarians, farmers, livestock transporters, slaughterhouse workers, butchers, hunters, furriers and wildlife carers (D'Amelio et al. 2015; Dutkiewicz et al. 2011; Kosevska et al. 2010). Foxes have provided meat and fur for humans since ancient times (Blank and Li 2021; Monchot and Gendron 2011), and some fox species continue to be harvested for these purposes to this day. While sales of animal fur have declined steeply in recent years due to the influence of animal welfare concerns on consumer preferences (Achabou et al. 2020; Halliday and McCulloch 2022) and the banning of fur farming in several formerly permissive countries (Arney 2022), farmed fur remains a lucrative global industry. Reported statistics, however, are often incomplete or conflicting. In 2023, active fur farming countries reportedly included Canada, China, Denmark, Iceland, Italy, Latvia, Lithuania, Poland, Romania and Russia (Ritchie et al. 2024; Warwick et al. 2023). There were an estimated 1000 active fur farms in the European Union alone, containing approximately 7.7 million animals—mainly mink, fox and raccoon dogs (EC 2023). One online database valued the global trade in raw fox furskins—most commonly from red or ‘silver’ foxes (*Vulpes vulpes*) and Arctic or ‘blue’ foxes (*Vulpes lagopus*)—at $70.9 million USD in 2022, with Finland accounting for over 90% of the export market (OEC 2024). China reportedly produced 12.53 million fox skins in 2020 (CLIA 2021). Fox farming is an intensive agricultural practice; individual farms hold large numbers of animals in close confinement, being fed abnormal diets to favour rapid growth (Warwick et al. 2023). Such conditions can impair animal health and promote emergence, mutation and spillover of infectious diseases, including zoonoses—as was highlighted during the SARS‐CoV‐2 pandemic, with spillover and reverse spillover events in mink farms facilitating viral mutations and leading to the cull of 17 million mink in Denmark alone (Dyer 2020; Jahid et al. 2024). A comprehensive study of viruses in Chinese fur animals identified arctic foxes as carriers of thirteen zoonotic virus species from six viral families, ranking them among the highest‐risk host species for potential cross‐species transmission (Zhao et al. 2024).

Exposure to fox‐origin zoonoses may still occur in settings considered low risk or lacking obvious risk factors. A review of 23 UK media reports describing unprovoked fox attacks on humans found 16 incidents of foxes entering homes and gardens and biting children and adults, most of whom were asleep at the time, and one case of a woman who was bitten on the ankle while walking to work in London (Bridge and Harris 2020). The abundance of anthropogenic resources and absence of natural predators in urban areas may be increasing the boldness of foxes in some settings (Padovani et al. 2021). Additionally, a recent study suggests that infection with the widely distributed parasite, *Toxoplasma gondii*, may be causing or contributing to ‘dopey fox syndrome’, in which affected foxes exhibit a range of aberrant behaviours, including reduced fear of humans (Milne et al. 2020).

Given the risk of transmission of zoonotic diseases and other injuries from foxes to humans, and the increasing overlap between fox and human habitats due to urbanisation and land‐use changes, proactive general protective strategies should be implemented across all settings where foxes are present. Large‐scale fox control or reduction is not a realistic option; such programs would be complex and require extensive concerted and sustained inputs from numerous sectors, and may face community opposition. Effects of lethal control programs are also typically short‐lived, with studies showing fox populations quickly rebound, most likely via immigration from surrounding areas and/or compensatory reproduction (Baker and Harris 2006; Kämmerle et al. 2019; Newsome et al. 2014). Furthermore, some native fox species are protected wildlife, such as the endangered San Joaquin kit fox (*Vulpes macrotis mutica*) and island fox (*Urocyon littoralis*) that are native to certain parts of the USA (Bjurlin and Cypher 2005; Wittmer et al. 2013).

Community education and outreach programs may be beneficial in discouraging people from seeking contact with foxes and thereby decrease the risk of zoonotic transmission of bacterial and other pathogens. Basic biosecurity measures such as hand washing, prompt cleaning and disinfection of any bites or scratches, and the use of protective personal equipment such as gloves would be recommended for people who handle foxes. While established biosecurity guidelines exist for preventing infectious disease introduction from humans and other animals into farmed fur animal populations (NFACC 2013; OIE 2021), there seems to be a lack of similar guidance specifically addressing the protection of human workers in those settings. Given the confluence of conditions favouring zoonotic disease transfer in commercial fur farming operations, the development of biosafety guidelines for human health protection in the fur animal industry is strongly recommended. These could be adapted from long‐established guidelines used in high‐risk occupational settings, including laboratories, animal facilities, and the wildlife trade (CDC 2020; OIE 2018; WOAH 2024).

Food hygiene measures, including proper dressing and cooking of game meat (AVMA 2024; USDA 2017), would be recommended for hunters and consumers of wild animals. Many zoonotic infections are foodborne, and while nationally legislated meat processing and inspection practices remove many pathogens from commercial food chains (FAO 2019; FAO and WHO 2005; OIE 2022; Young et al. 2016), individuals may still hunt and consume game meat that is not subjected to these safeguards. While fox meat is not typically consumed in most societies, there are some exceptions. People of the Kazakh ethnic minority in Mongolia, the Hadzabe tribe from Tanzania, and various indigenous groups from northern North America are reported to supplement their normal diet with fox meat; some from preference and others out of necessity during periods of scarcity (Kuhnlein and Humphries 2017; Mohan 2015; Sharon 2023). One Chinese article reportedly describes a novel technological process for producing processed meat products, including bacon and sausages, from fox and raccoon dog meats as a productive use of fur animal byproducts (Zuo et al. 2003), while the sale of fox meat from Scandinavian fur farms in a butcher shop in Gravesend, UK in 2011, purportedly sparked outrage from local and online communities (Holmberg 2011). Unintentional consumption of fox meat has also been known to occur: the 2014 ‘fox meat scandal’ saw mega‐retailer Walmart in China recall 'five spice' donkey meat products—reportedly a popular snack in some parts of the country—after laboratory testing showed contamination with fox meat (Jourdan 2014; Whitehead 2014). Whether intentional or deliberate, consumption of fox meat carries an inherent risk of exposure to zoonotic bacteria, viruses and parasites, and consumers should implement proper food handling and cooking practices to mitigate infection risk.

Our scoping review has some limitations. The heterogeneity of the data extracted from the examined articles, including differences in study designs, locations, populations, and testing methods, prevented meaningful in‐depth comparisons or interpretations of cross‐cutting themes or trends. While we extensively pre‐tested and refined our search strategy prior to implementation, and implemented the searches in five databases, some relevant articles may have been missed. We could only review studies for which the full text, in English, was available; several potentially relevant articles published in other languages were excluded during the screening steps. Although the inter‐rater reliability rating for our title and abstract screening round was relatively low, all discordance was resolved by consensus and assessment of evidence against the inclusion criteria. Our experience underscores the need for complete secondary screening for these kinds of scoping reviews, where the diversity of study designs and outputs increases the complexity of initial title and abstract screening.

## 5 Conclusions

This scoping review systematically searched the global literature and identified ten published reports investigating fox‐human transmission of zoonotic bacteria. Both the small total number of published articles and the typically low reported likelihood of fox‐origin transmission in those examined articles suggest there is limited evidence of foxes transmitting zoonotic bacteria to humans. However, our data confirm that transmission risks do exist, especially where human behaviours facilitate human‐fox interactions such as high‐risk occupational settings and geographical areas with established populations of increasingly human‐habituated foxes. Recommendations include integrated One Health approaches to further investigate carriage and transmission of zoonotic bacteria in foxes, including publication of negative findings, and mitigation of zoonotic disease risk via educational public health campaigns and the implementation of human protective measures.

## Author Contributions

Concept idea: All; Drafting protocol: E.C.H., M.M. and B.M.; Defining eligibility criteria: E.C.H., M.M., B.M. and S.H.; Search strategy: S.H., E.C.H., M.M. and B.M.; Search verification: S.H., E.C.H., M.M. and B.M.; Title and abstract screening; E.C.H., M.M. and B.M.; Full‐text screening: E.C.H., M.M. and B.M.; Data analysis and synthesis of results: E.C.H., M.M. and B.M.; Drafting paper: E.C.H., M.M. and B.M.; Reviewing paper: All.

## Conflicts of Interest

The authors declare no conflicts of interest.

## Supporting information


**Appendix S1.** ‘PRISMA for scoping reviews (PRISMA‐ScR) checklist’ (Tricco et al. 2018) completed for this scoping review.

**Appendix S2.** Database search strategies utilised for this scoping review.

**Appendix S3.** Instructions for screeners.

**Appendix S4.** Data extraction template.

**Appendix S5.** Extracted data from the 10 studies examined for this scoping review.

## Data Availability

The data that supports the findings of this study are available in the supplementary material of this article.

## References

[zph13230-bib-0001] Achabou, M. A. , S. Dekhili , and A. P. Codini . 2020. “Consumer Preferences Towards Animal‐Friendly Fashion Products: An Application to the Italian Market.” Journal of Consumer Marketing 37, no. 6: 661–673. 10.1108/JCM-10-2018-2908.

[zph13230-bib-0002] Aikembayev, A. M. , L. Lukhnova , G. Temiraliyeva , et al. 2010. “Historical Distribution and Molecular Diversity of *Bacillus Anthracis*, Kazakhstan.” Emerging Infectious Diseases 16, no. 5: 789–796. 10.3201/eid1605.091427. 20409368 PMC2953997

[zph13230-bib-0003] Allen, T. , K. A. Murray , C. Zambrana‐Torrelio , et al. 2017. “Global Hotspots and Correlates of Emerging Zoonotic Diseases.” Nature Communications 8, no. 1: 1124. 10.1038/s41467-017-00923-8. PMC5654761 29066781

[zph13230-bib-0004] Arney, D. 2022. “Farming Non‐Domesticated and Semi‐Domesticated Terrestrial Species.” In Routledge Handbook of Animal Welfare, edited by A. Knight , C. Phillips , and P. Sparks , 103–114. Routledge. 10.4324/9781003182351-11.

[zph13230-bib-0005] AVMA . 2024. “Disease Precautions for Hunters.” https://www.avma.org/resources/public‐health/disease‐precautions‐hunters.

[zph13230-bib-0006] Baker, P. J. , S. M. Funk , S. A. Harris , T. J. Newman , G. R. Saunders , and P. C. L. White . 2004. The Impact of Human Attitudes on the Social and Spatial Organisation of Urban Foxes (*Vulpes Vulpes*) Before and After an Outbreak of Sarcoptic Mange Paper presented at the 4th International Symposium on Urban Wildlife Conservation, Arizona, USA.

[zph13230-bib-0007] Baker, P. J. , and S. Harris . 2006. “Does Culling Reduce Fox (*Vulpes vulpes*) Density in Commercial Forests in Wales, UK?” European Journal of Wildlife Research 52, no. 2: 99–108. 10.1007/s10344-005-0018-y.

[zph13230-bib-0008] Baker, P. J. , T. J. Newman , and S. Harris . 2001. “Bristol's Foxes–40 Years of Change.” British Wildlife 12, no. 6: 411–418.

[zph13230-bib-0009] Bateman, P. W. , and P. A. Fleming . 2012. “Big City Life: Carnivores in Urban Environments.” Journal of Zoology 287, no. 1: 1–23. 10.1111/j.1469-7998.2011.00887.x.

[zph13230-bib-0010] Bjurlin, C. D. , and B. L. Cypher . 2005. “Encounter Frequency With the Urbanized San Joaquin Kit Fox Correlates With Public Beliefs and Attitudes Toward the Species.” Endangered Species Update 22: 107–115.

[zph13230-bib-0011] Blank, D. , and Y. Li . 2021. “Sustainable Use of Wildlife Resources in Central Asia.” Regional Sustainability 2, no. 2: 144–155. 10.1016/j.regsus.2021.05.001.

[zph13230-bib-0012] Blasdell, K. R. , B. McNamara , D. P. O'Brien , et al. 2022. “Environmental Risk Factors Associated With the Presence of *Mycobacterium Ulcerans* in Victoria, Australia.” PLoS One 17, no. 9: e0274627. 10.1371/journal.pone.0274627. 36099259 PMC9469944

[zph13230-bib-0013] Bordes, L. , S. Vreman , R. Heutink , et al. 2023. “Highly Pathogenic Avian Influenza H5N1 Virus Infections in Wild Red Foxes (*Vulpes Vulpes*) Show Neurotropism and Adaptive Virus Mutations.” Microbiology Spectrum 11, no. 1: e0286722. 10.1128/spectrum.02867-22. 36688676 PMC9927208

[zph13230-bib-0014] Bridge, B. , and S. Harris . 2020. “Do Urban Red Foxes Attack People? An Exploratory Study and Review of Incidents in Britain.” Human‐Wildlife Interactions 14, no. 2: 151–165. 10.26077/d6f5-f6f3.

[zph13230-bib-0015] Buultjens, A. H. , K. Vandelannoote , C. J. Meehan , et al. 2018. “Comparative Genomics Shows That *Mycobacterium ulcerans* Migration and Expansion Preceded the Rise of Buruli Ulcer in Southeastern Australia.” Applied and Environmental Microbiology 84, no. 8: e02612–e02617. 10.1128/aem.02612-17. 29439984 PMC5881063

[zph13230-bib-0016] Carter, A. , G. W. Luck , and S. P. McDonald . 2012. “Ecology of the Red Fox (*Vulpes Vulpes*) in an Agricultural Landscape. 2. Home Range and Movements.” Australian Mammalogy 34, no. 2: 175–187. 10.1071/AM11041.

[zph13230-bib-0017] Castañeda, I. , T. S. Doherty , P. A. Fleming , A. M. Stobo‐Wilson , J. C. Z. Woinarski , and T. M. Newsome . 2022. “Variation in Red Fox *Vulpes Vulpes* Diet in Five Continents.” Mammal Review 52, no. 3: 328–342. 10.1111/mam.12292.

[zph13230-bib-0018] CDC . 2020. “Biosafety in Microbiological and Biomedical Laboratories.” https://www.cdc.gov/labs/bmbl/index.html.

[zph13230-bib-0019] Christie, A. B. 1981. “Rabies.” Journal of Infection 3, no. 3: 202–218. 10.1016/S0163-4453(81)90750-7. 6764492

[zph13230-bib-0020] Christou, L. 2011. “The Global Burden of Bacterial and Viral Zoonotic Infections.” Clinical Microbiology and Infection 17, no. 3: 326–330. 10.1111/j.1469-0691.2010.03441.x. 21129102 PMC7129620

[zph13230-bib-0021] CLIA . 2021. “Statistical Report on the Production of Skins of Mink, Fox and Raccoon in China (2020).” https://www.chinaleather.org/front/article/115225/366.

[zph13230-bib-0022] Cohen, J. 1960. “A Coefficient of Agreement for Nominal Scales.” Educational and Psychological Measurement 20, no. 1: 37–46. 10.1177/001316446002000104.

[zph13230-bib-0023] Conraths, F. J. , C. Probst , A. Possenti , et al. 2017. “Potential Risk Factors Associated With Human Alveolar Echinococcosis: Systematic Review and Meta‐Analysis.” PLoS Neglected Tropical Diseases 11, no. 7: e0005801. 10.1371/journal.pntd.0005801. 28715408 PMC5531747

[zph13230-bib-0024] Contesse, P. , D. Hegglin , S. Gloor , F. Bontadina , and P. Deplazes . 2004. “The Diet of Urban Foxes (*Vulpes Vulpes*) and the Availability of Anthropogenic Food in the City of Zurich, Switzerland.” Mammalian Biology 69, no. 2: 81–95. 10.1078/1616-5047-00123.

[zph13230-bib-0025] Craig, P. 2003. “ Echinococcus multilocularis .” Current Opinion in Infectious Diseases 16, no. 5: 437–444. 10.1097/00001432-200310000-00010. 14501996

[zph13230-bib-0026] Crits‐Christoph, A. , J. I. Levy , J. E. Pekar , et al. 2024. “Genetic Tracing of Market Wildlife and Viruses at the Epicenter of the COVID‐19 Pandemic.” Cell 187, no. 19: 5468–5482. 10.1016/j.cell.2024.08.010. 39303692 PMC11427129

[zph13230-bib-0027] Cuarón, A. D. , M. A. Martínez‐Morales , K. W. McFadden , D. Valenzuela , and M. E. Gompper . 2004. “The Status of Dwarf Carnivores on Cozumel Island, Mexico.” Biodiversity and Conservation 13, no. 2: 317–331. 10.1023/b:bioc.0000006501.80472.cc.

[zph13230-bib-0028] D'Amelio, E. , B. Gentile , F. Lista , and R. D'Amelio . 2015. “Historical Evolution of Human Anthrax From Occupational Disease to Potentially Global Threat as Bioweapon.” Environment International 85: 133–146. 10.1016/j.envint.2015.09.009. 26386727

[zph13230-bib-0029] Dickman, C. R. 1996. “Impact of Exotic Generalist Predators on the Native Fauna of Australia.” Wildlife Biology 2, no. 3: 185–195. 10.2981/wlb.1996.018.

[zph13230-bib-0030] Dumas, D. , and R. Touma . 2024. “Outfoxed: The ‘Smart’ Ferals Are Adapting to Australian Cities, and Wreaking Havoc in the Bush, Html.” https://www.theguardian.com/environment/article/2024/jul/15/australia‐foxes‐risks‐native‐flora‐fauna.

[zph13230-bib-0031] Dutkiewicz, J. , E. Cisak , J. Sroka , A. Wójcik‐Fatla , and V. Zając . 2011. “Biological Agents as Occupational Hazards—Selected Issues.” Annals of Agricultural and Environmental Medicine 18, no. 2: 286–293. 22216801

[zph13230-bib-0032] Dyer, O. 2020. “Covid‐19: Denmark to Kill 17 Million Minks Over Mutation That Could Undermine Vaccine Effort.” British Medical Journal 371: m4338. 10.1136/bmj.m4338. 33168526

[zph13230-bib-0033] EC . 2023. “Questions and Answers on the European Citizens' Initiative “Fur Free Europe”.” https://ec.europa.eu/commission/presscorner/detail/en/qanda_23_6254.

[zph13230-bib-0034] Ervin, J. S. , L. C. Van De Werfhorst , J. L. S. Murray , and P. A. Holden . 2014. “Microbial Source Tracking in a Coastal California Watershed Reveals Canines as Controllable Sources of Fecal Contamination.” Environmental Science and Technology 48, no. 16: 9043–9052. 10.1021/es502173s. 25055204

[zph13230-bib-0035] Esmaeili, S. , M. M. Gooya , M. R. Shirzadi , et al. 2014. “Seroepidemiological Survey of Tularemia Among Different Groups in Western Iran.” International Journal of Infectious Diseases 18: 27–31. 10.1016/j.ijid.2013.08.013. 24145011

[zph13230-bib-0036] FAO . 2019. “Technical Guidance Principles of Risk‐Based Meat Inspection and Their Application.” https://openknowledge.fao.org/server/api/core/bitstreams/24867604‐68dd‐4652‐ac42‐32825e8b34fa/content.

[zph13230-bib-0037] FAO , and WHO . 2005. “Code of Hygienic Practice for Meat.” In Codex Alimentarus. Food and Agriculture Organization of the United Nations (UN) and the World Health Organization (WHO). https://www.fao.org/fao‐who‐codexalimentarius/codex‐texts/codes‐of‐practice/en/.

[zph13230-bib-0038] Fleming, P. A. , A. M. Stobo‐Wilson , H. M. Crawford , et al. 2022. “Distinctive Diets of Eutherian Predators in Australia.” Royal Society Open Science 9, no. 10: 220792. 10.1098/rsos.220792. 36312571 PMC9554524

[zph13230-bib-0039] Fooks, A. R. , F. Cliquet , S. Finke , et al. 2017. “Rabies.” Nature Reviews Disease Primers 3, no. 1: 17091. 10.1038/nrdp.2017.91. 29188797

[zph13230-bib-0040] Fyfe, J. A. M. , C. J. Lavender , K. A. Handasyde , et al. 2010. “A Major Role for Mammals in the Ecology of *Mycobacterium ulcerans* .” PLoS Neglected Tropical Diseases 4, no. 8: e791. 10.1371/journal.pntd.0000791. 20706592 PMC2919402

[zph13230-bib-0041] Gallagher, G. , S. Mann , and F. Sundram . 2013. “Weil's Disease After a Fox Bite in Inner City London.” Paper Presented at the 23rd Conference of the Asian Pacific Association for the Study of the Liver, Singapore. https://link.springer.com/article/10.1007/s12072‐013‐9429‐0.

[zph13230-bib-0042] Grace, D. , F. Mutua , P. Ochungo , et al. 2012. “Mapping of Poverty and Likely Zoonoses Hotspots.” https://cgspace.cgiar.org/handle/10568/21161.

[zph13230-bib-0043] Guthrie, A. 2019. “85% Unaware That Encouraging Foxes Might Spread Lungworm to Pet Dogs.” https://www.vetsurgeon.org/b/veterinary‐news/posts/85‐unaware‐that‐encouraging‐foxes‐might‐spread‐lungworm‐to‐pet‐dogs.

[zph13230-bib-0044] Halliday, C. , and S. P. McCulloch . 2022. “Beliefs and Attitudes of British Residents About the Welfare of Fur‐Farmed Species and the Import and Sale of Fur Products in the UK.” Animals 12, no. 5: 538. 10.3390/ani12050538. 35268109 PMC8908824

[zph13230-bib-0045] Harper, M. J. 2005. “Home Range and Den Use of Common Brushtail Possums (*Trichosurus vulpecula*) in Urban Forest Remnants.” Wildlife Research 32, no. 8: 681. 10.1071/wr04072.

[zph13230-bib-0046] Heydon, M. J. , J. C. Reynolds , and M. J. Short . 2000. “Variation in Abundance of Foxes (*Vulpes vulpes*) Between Three Regions of Rural Britain, in Relation to Landscape and Other Variables.” Journal of Zoology 251, no. 2: 253–264. 10.1111/j.1469-7998.2000.tb00608.x.

[zph13230-bib-0047] Hobbs, E. C. , J. L. Porter , J. Y. H. Lee , et al. 2024. “Buruli Ulcer Surveillance in South‐Eastern Australian Possums: Infection Status, Lesion Mapping and Internal Distribution of *Mycobacterium ulcerans* .” PLoS Neglected Tropical Diseases 18, no. 11: e0012189. 10.1371/journal.pntd.0012189. 39499725 PMC11581399

[zph13230-bib-0048] Holland, C. V. , Z. G. Afra , S. Valizadeh , M. Ebrahimi , and A. Rostami . 2024. “The Global Prevalence of *Toxocara Canis* Among Red Foxes (*Vulpes Vulpes*): A Systematic Review and Meta‐Analysis.” International Journal for Parasitology: Parasites and Wildlife 25: 100984. 10.1016/j.ijppaw.2024.100984. 39297146 PMC11409046

[zph13230-bib-0049] Holmberg, A. 2011. “Danish Foxes on Dinner Tables in England.” https://www.change.org/p/danish‐foxes‐on‐dinner‐tables‐in‐england.

[zph13230-bib-0050] Horby, P. W. , N. T. Hoa , D. U. Pfeiffer , and H. F. L. Wertheim . 2014. “Drivers of Emerging Zoonotic Infectious Diseases.” In Confronting Emerging Zoonoses: The One Health Paradigm, edited by A. Yamada , L. Kahn , B. Kaplan , T. Monath , J. Woodall , and L. Conti , 13–26. Springer Japan. 10.1007/978-4-431-55120-1_2.

[zph13230-bib-0051] IUCN . 2024. “The IUCN Red List of Threatened Species.” https://www.iucnredlist.org/.

[zph13230-bib-0052] Jahid, M. J. , A. S. Bowman , and J. M. Nolting . 2024. “SARS‐CoV‐2 Outbreaks on Mink Farms—A Review of Current Knowledge on Virus Infection, Spread, Spillover, and Containment.” Viruses 16, no. 1: 81. 10.3390/v16010081. 38257781 PMC10819236

[zph13230-bib-0053] Johnson, P. D. R. , J. Azuolas , C. J. Lavender , et al. 2007. “ *Mycobacterium ulcerans* in Mosquitoes Captured During Outbreak of Buruli Ulcer, Southeastern Australia.” Emerging Infectious Diseases 13, no. 11: 1653–1660. 10.3201/eid1311.061369. 18217547 PMC3375796

[zph13230-bib-0054] Jones, K. E. , N. G. Patel , M. A. Levy , et al. 2008. “Global Trends in Emerging Infectious Diseases.” Nature 451, no. 7181: 990–993. 10.1038/nature06536. 18288193 PMC5960580

[zph13230-bib-0055] Jourdan, A. 2014. “Wal‐Mart Recalls Donkey Product in China After Fox Meat Scandal, Html. Reuters.” https://tinyurl.com/3j8excar.

[zph13230-bib-0056] Kamler, J. F. , and W. B. Ballard . 2002. “A Review of Native and Nonnative Red Foxes in North America.” Wildlife Society Bulletin (1973–2006) 30, no. 2: 370–379.

[zph13230-bib-0057] Kämmerle, J. , E. G. Ritchie , and I. Storch . 2019. “Restricted‐Area Culls and Red Fox Abundance: Are Effects a Matter of Time and Place?” Conservation Science and Practice 1, no. 11: e115. 10.1111/csp2.115.

[zph13230-bib-0058] Karamon, J. , J. Dąbrowska , M. Kochanowski , et al. 2018. “Prevalence of Intestinal Helminths of Red Foxes (*Vulpes vulpes*) in Central Europe (Poland): A Significant Zoonotic Threat.” Parasites & Vectors 11, no. 1: 436. 10.1186/s13071-018-3021-3. 30055657 PMC6064108

[zph13230-bib-0059] Karayiannis, S. , P. Ntais , I. Messaritakis , N. Tsirigotakis , E. Dokianakis , and M. Antoniou . 2015. “Detection of *Leishmania Infantum* in Red Foxes (*Vulpes vulpes*) in Central Greece.” Parasitology 142, no. 13: 1574–1578. 10.1017/S0031182015001158. 26399545

[zph13230-bib-0060] Karesh, W. B. , A. Dobson , J. O. Lloyd‐Smith , et al. 2012. “Ecology of Zoonoses: Natural and Unnatural Histories.” Lancet 380, no. 9857: 1936–1945. 10.1016/s0140-6736(12)61678-x. 23200502 PMC7138068

[zph13230-bib-0061] Kobryn, H. T. , E. J. Swinhoe , P. W. Bateman , P. J. Adams , J. M. Shephard , and P. A. Fleming . 2023. “Foxes at Your Front Door? Habitat Selection and Home Range Estimation of Suburban Red Foxes (*Vulpes vulpes*).” Urban Ecosystems 26, no. 1: 1–17. 10.1007/s11252-022-01252-5.

[zph13230-bib-0062] Kosevska, E. , D. Donev , G. Kuzmanovska , V. Karamandi‐Lazarovska , and S. Pitlichkovska . 2010. “Health Promotion and Prevention of Human Brucellosis in the Republic of Macedonia.” Macedonian Journal of Medical Sciences 3, no. 3: 283–288. 10.3889/MJMS.1857-5773.2010.0136.

[zph13230-bib-0063] Kuhnlein, H. V. , and M. M. Humphries . 2017. Fox. Traditional Animal Foods of Indigenous Peoples of Northern North America. McGill University. http://traditionalanimalfoods.org/mammals/furbearers/page.aspx?id=6367.

[zph13230-bib-0064] Lavender, C. J. , J. A. M. Fyfe , J. Azuolas , et al. 2011. “Risk of Buruli Ulcer and Detection of *Mycobacterium ulcerans* in Mosquitoes in Southeastern Australia.” PLoS Neglected Tropical Diseases 5, no. 9: e1305. 10.1371/journal.pntd.0001305. 21949891 PMC3176747

[zph13230-bib-0065] Lindsø, L. K. , P. Dupont , L. Rød‐Eriksen , et al. 2022. “Estimating Red Fox Density Using Non‐Invasive Genetic Sampling and Spatial Capture‐Recapture Modelling.” Oecologia 198, no. 1: 139–151. 10.1007/s00442-021-05087-3. 34859281 PMC8803778

[zph13230-bib-0066] Marks, C. A. , and T. E. Bloomfield . 1999. “Distribution and Density Estimates for Urban Foxes (*Vulpes vulpes*) in Melbourne: Implications for Rabies Control.” Wildlife Research 26, no. 6: 763. 10.1071/wr98059.

[zph13230-bib-0067] McNamara, B. , J. Cornish , K. R. Blasdell , et al. 2025. “ *Mycobacterium ulcerans* in Possum Feces Before Emergence in Humans, Australia.” Emerging Infectious Diseases 31, no. 3: 569–573. https://pubmed.ncbi.nlm.nih.gov/40023807/. 10.3201/eid3103.240657. 40023807 PMC11878303

[zph13230-bib-0068] Mee, P. T. , A. H. Buultjens , J. Oliver , et al. 2024. “Mosquitoes Provide a Transmission Route Between Possums and Humans for Buruli Ulcer in Southeastern Australia.” Nature Microbiology 9: 377–389. 10.1038/s41564-023-01553-1. PMC10847040 38263454

[zph13230-bib-0069] Meyers, W. M. , B. J. Gormus , G. P. Walsh , G. B. Baskin , and G. B. Hubbard . 1991. “Naturally Acquired and Experimental Leprosy in Nonhuman Primates.” American Journal of Tropical Medicine and Hygiene 44, no. 4: 24–27. 10.4269/ajtmh.1991.44.24. 2042709

[zph13230-bib-0070] Milne, G. , C. Fujimoto , T. Bean , et al. 2020. “Infectious Causation of Abnormal Host Behavior: *Toxoplasma Gondii* and Its Potential Association With Dopey Fox Syndrome.” Frontiers in Psychiatry 11: 513536. 10.3389/fpsyt.2020.513536. 33192643 PMC7525129

[zph13230-bib-0071] Mohan, P. 2015. Hunting With Eagles: In the Realm of the Mongolian Kazakhs. Merrell Publishers Ltd.

[zph13230-bib-0072] Mohebali, M. , E. Moradi‐Asl , and Y. Rassi . 2018. “Geographic Distribution and Spatial Analysis of *Leishmania Infantum* Infection in Domestic and Wild Animal Reservoir Hosts of Zoonotic Visceral Leishmaniasis in Iran: A Systematic Review.” Journal of Vector Borne Diseases 55, no. 3: 173–183. 10.4103/0972-9062.249125. 30618442

[zph13230-bib-0073] Monchot, H. , and D. Gendron . 2011. “Fox Exploitation by the Paleoeskimo at the Tayara Site, Nunavik.” Arctic Anthropology 48, no. 1: 15–32.

[zph13230-bib-0074] Moore, L. S. P. , A. Leslie , M. Meltzer , A. Sandison , A. Efstratiou , and S. Sriskandan . 2015. “ *Corynebacterium ulcerans* Cutaneous Diphtheria.” Lancet Infectious Diseases 15, no. 9: 1100–1107. 10.1016/s1473-3099(15)00225-x. 26189434

[zph13230-bib-0075] Newsome, T. M. , M. S. Crowther , and C. R. Dickman . 2014. “Rapid Recolonisation by the European Red Fox: How Effective Are Uncoordinated and Isolated Control Programs?” European Journal of Wildlife Research 60, no. 5: 749–757. 10.1007/s10344-014-0844-x.

[zph13230-bib-0076] Neyra, R. C. , J. A. Frisancho , J. L. Rinsky , et al. 2014. “Multidrug‐Resistant and Methicillin‐Resistant *Staphylococcus aureus* (MRSA) in Hog Slaughter and Processing Plant Workers and Their Community in North Carolina (USA).” Environmental Health Perspectives 122, no. 5: 471–477. 10.1289/ehp.1306741. 24508836 PMC4014760

[zph13230-bib-0077] NFACC . 2013. “Code of Practice for the Care and Handling of Farmed Fox (Vulpes Vulpes) (978–1–988793‐22‐1 (Electronic Version)).” https://www.nfacc.ca/pdfs/codes/Farmed_Fox_Code.pdf.

[zph13230-bib-0078] Nurumal, S. R. , J. Mansor , M. Ghazali , et al. 2022. “Animal Rabies: A Systematic Review.” Malaysian Journal of Public Health Medicine 22, no. 3: 145–152. 10.37268/mjphm/vol.22/no.3/art.1258.

[zph13230-bib-0079] O'Brien, C. R. , K. A. Handasyde , J. Hibble , et al. 2014. “Clinical, Microbiological and Pathological Findings of *Mycobacterium ulcerans* Infection in Three Australian Possum Species.” PLoS Neglected Tropical Diseases 8, no. 1: e2666. 10.1371/journal.pntd.0002666. 24498451 PMC3907337

[zph13230-bib-0080] OEC . 2024. “Raw Fox Furskins, Whole. v5.0.” https://oec.world/en/profile/hs/raw‐fox‐furskins‐whole.

[zph13230-bib-0081] OIE . 2018. “Biosafety and Biosecurity: Standard for Managing Biological Risk in the Veterinary Laboratory and Animal Facilities.” In OIE Terrestrial Manual, 16. World Organisation for Animal Health (OIE). https://www.woah.org/fileadmin/Home/eng/Health_standards/tahm/1.01.04_BIOSAFETY_BIOSECURITY.pdf.

[zph13230-bib-0082] OIE . 2021. “Guidance on Working With Farmed Animals of Species Susceptible to Infection With SARS‐CoV‐2.” https://www.woah.org/app/uploads/2021/06/en‐oie‐guidance‐farmed‐animals.pdf.

[zph13230-bib-0083] OIE . 2022. “Control of Biological Hazards of Animal Health and Public Health Importance Through Ante‐ and Post‐Mortem Meat Inspection.” In OIE Terrestrial Animal Health Code. World Organisation for Animal Health (OIE). https://www.woah.org/fileadmin/Home/eng/Health_standards/tahc/current/chapitre_control_bio_hazard.pdf.

[zph13230-bib-0084] Oksanen, A. , M. Siles‐Lucas , J. Karamon , et al. 2016. “The Geographical Distribution and Prevalence of *Echinococcus Multilocularis* in Animals in the European Union and Adjacent Countries: A Systematic Review and Meta‐Analysis.” Parasites & Vectors 9, no. 1: 519. 10.1186/s13071-016-1746-4. 27682156 PMC5039905

[zph13230-bib-0085] Padovani, R. , Z. Shi , and S. Harris . 2021. “Are British Urban Foxes (*Vulpes vulpes*) “Bold”? The Importance of Understanding Human–Wildlife Interactions in Urban Areas.” Ecology and Evolution 11, no. 2: 835–851. 10.1002/ece3.7087. 33520170 PMC7820170

[zph13230-bib-0086] Perera, J. M. , C. Gurtler , and A. N. Barnes . 2024. “A Systematic Review of Zoonotic Enteric Parasites in Synanthropic Mammalian Species in Florida.” Pathogens 13, no. 12: 1065. https://www.mdpi.com/2076‐0817/13/12/1065. 10.3390/pathogens13121065. 39770325 PMC11728782

[zph13230-bib-0087] Peters, M. D. J. , C. Godfrey , P. McInerney , Z. Munn , A. C. Tricco , and H. Khalil . 2020. “Chapter 11: Scoping Reviews.” In JBI Manual for Evidence Synthesis. edited by E. Aromataris and Z. Munn . JBI Global. https://synthesismanual.jbi.global/, 10.46658/JBIMES-20-12.

[zph13230-bib-0088] Porter, S. M. , A. E. Hartwig , H. Bielefeldt‐Ohmann , A. M. Bosco‐Lauth , and J. J. Root . 2022. “Susceptibility of Wild Canids to SARS‐CoV‐2.” Emerging Infectious Diseases 28, no. 9: 1852–1855. 10.3201/eid2809.220223. 35830965 PMC9423904

[zph13230-bib-0089] Rahman, M. T. , M. A. Sobur , M. S. Islam , et al. 2020. “Zoonotic Diseases: Etiology, Impact, and Control.” Microorganisms 8, no. 9: 1405. 10.3390/microorganisms8091405. 32932606 PMC7563794

[zph13230-bib-0090] Ritchie, H. , P. Rosado , and M. Roser . 2024. “Data Page: Active Fur Farms (Online Resource).” https://ourworldindata.org/grapher/active‐fur‐farms.

[zph13230-bib-0091] Saunders, G. R. , M. N. Gentle , and C. R. Dickman . 2010. “The Impacts and Management of Foxes *Vulpes vulpes* in Australia.” Mammal Review 40, no. 3: 181–211. 10.1111/j.1365-2907.2010.00159.x.

[zph13230-bib-0092] Schilling, A.‐K. , C. Avanzi , R. G. Ulrich , et al. 2019. “British Red Squirrels Remain the Only Known Wild Rodent Host for Leprosy Bacilli.” Frontiers in Veterinary Science 6: 8. 10.3389/fvets.2019.00008. 30775369 PMC6367869

[zph13230-bib-0093] Scott, D. M. , M. J. Berg , B. A. Tolhurst , et al. 2014. “Changes in the Distribution of Red Foxes (*Vulpes vulpes*) in Urban Areas in Great Britain: Findings and Limitations of a Media‐Driven Nationwide Survey.” PLoS One 9, no. 6: e99059. 10.1371/journal.pone.0099059. 24919063 PMC4053368

[zph13230-bib-0094] Sharma, R. , P. Singh , W. J. Loughry , et al. 2015. “Zoonotic Leprosy in the Southeastern United States.” Emerging Infectious Diseases 21, no. 12: 2127–2134. 10.3201/eid2112.150501. 26583204 PMC4672434

[zph13230-bib-0095] Sharon, F. 2023. “Why Hadzabe Tribe Like to Eat Wild Fox Meat for Dinner? See How They Hunt and Their Recipes.”

[zph13230-bib-0096] Silva, F. , L. Mathias , S. Loffer , et al. 2015. “Isolation of Leptospira spp. in Small Farm Populations in Nine States of Brazil.” International Journal of Epidemiology 44: i204. 10.1093/ije/dyv096.333.

[zph13230-bib-0097] Silva, F. J. , C. E. P. Santos , G. C. P. Silva , R. F. Santos , V. C. M. Curci , and L. A. Mathias . 2014. “The Importance of *Leptospira Interrogans* Serovars *Icterohaemorrhagiae* and *Canicola* in Coastal Zone and in Southern Fields of Rio Grande Do Sul, Brazil.” Pesquisa Veterinaria Brasileira 34, no. 1: 34–38. 10.1590/S0100-736X2014000100006.

[zph13230-bib-0098] Tarek, M. H. , J. Hubbart , and E. Garner . 2023. “Microbial Source Tracking to Elucidate the Impact of Land‐Use and Physiochemical Water Quality on Fecal Contamination in a Mixed Land‐Use Watershed.” Science of the Total Environment 872: 162181. 10.1016/j.scitotenv.2023.162181. 36775177

[zph13230-bib-0099] Taylor, L. H. , S. M. Latham , and M. E. J. Woolhouse . 2001. “Risk Factors for Human Disease Emergence.” Philosophical Transactions of the Royal Society of London. Series B: Biological Sciences 356, no. 1411: 983–989. 10.1098/rstb.2001.0888. 11516376 PMC1088493

[zph13230-bib-0100] Tricco, A. C. , E. Lillie , W. Zarin , et al. 2018. “PRISMA Extension for Scoping Reviews (PRISMA‐ScR): Checklist and Explanation.” Annals of Internal Medicine 169, no. 7: 467–473. 10.7326/m18-0850. 30178033

[zph13230-bib-0101] Triggs, B. , H. Brunner , and J. Cullen . 1984. “The Food of Fox, Dog and Cat in Croajingalong National Park, South‐Eastern Victoria.” Wildlife Research 11, no. 3: 491–499. 10.1071/WR9840491.

[zph13230-bib-0102] USDA . 2017. “Roasting Those “Other” Holiday Meats.”

[zph13230-bib-0103] Veronesi, F. , G. Deak , and A. Diakou . 2023. “Wild Mesocarnivores as Reservoirs of Endoparasites Causing Important Zoonoses and Emerging Bridging Infections Across Europe.” Pathogens 12: 178. 10.3390/pathogens12020178. 36839450 PMC9964259

[zph13230-bib-0104] Warwick, C. , A. Pilny , C. Steedman , and R. Grant . 2023. “One Health Implications of Fur Farming.” Frontiers in Animal Science 4: 1249901. 10.3389/fanim.2023.1249901.

[zph13230-bib-0105] Whitehead, R. J. 2014. “Walmart Pulls Fox Meat Msquerading as Donkey Snacks.” Food Navigator Asia. https://www.foodnavigator‐asia.com/Article/2014/01/06/Walmart‐pulls‐fox‐meat‐masquerading‐as‐donkey‐snacks/.

[zph13230-bib-0106] WHO . 2023. “Number of New Reported Cases of Buruli Ulcer.” https://www.who.int/data/gho/data/indicators/indicator‐details/GHO/number‐of‐new‐reported‐cases‐of‐buruli‐ulcer.

[zph13230-bib-0107] Wittmer, H. U. , R. Serrouya , L. M. Elbroch , and A. J. Marshall . 2013. “Conservation Strategies for Species Affected by Apparent Competition.” Conservation Biology 27, no. 2: 254–260. 10.1111/cobi.12005. 23282104

[zph13230-bib-0108] WOAH . 2024. “Guidelines for Addressing Disease Risks in Wildlife Trade.” 10.20506/woah.3368. PMC11926677 40123917

[zph13230-bib-0109] Wojciech, Ł. , Z. Staroniewicz , A. Jakubczak , and M. Ugorski . 2004. “Typing of *Yersinia enterocolitica* Isolates by ITS Profiling, REP‐ and ERIC‐PCR.” Journal of Veterinary Medicine. B, Infectious Diseases and Veterinary Public Health 51, no. 5: 238–244. 10.1111/j.1439-0450.2004.00762.x. 15330984

[zph13230-bib-0110] Wolfe, N. D. , C. P. Dunavan , and J. Diamond . 2007. “Origins of Major Human Infectious Diseases.” Nature 447, no. 7142: 279–283. 10.1038/nature05775. 17507975 PMC7095142

[zph13230-bib-0111] Young, I. , B. J. Wilhelm , S. Cahill , R. Nakagawa , P. Desmarchelier , and A. Rajić . 2016. “A Rapid Systematic Review and Meta‐Analysis of the Efficacy of Slaughter and Processing Interventions to Control Nontyphoidal *Salmonella* in Beef and Pork.” Journal of Food Protection 79, no. 12: 2196–2210. 10.4315/0362-028X.JFP-16-203. 28104927 PMC5238939

[zph13230-bib-0112] Zhao, J. , W. Wan , K. Yu , et al. 2024. “Farmed Fur Animals Harbour Viruses With Zoonotic Spillover Potential.” Nature 634, no. 8032: 228–233. 10.1038/s41586-024-07901-3. 39232170 PMC11741233

[zph13230-bib-0113] Zuo, Y. , S. Zhao , C. Feng , X. Wu , B. Li , and X. Liu . 2003. “Processing of Fox and Racoon Dog Meats.” Journal of Economic Animal 7, no. 1: 10–12.

