# Supplementary material for: The Role of Foxes in Transmitting Zoonotic Bacteria to Humans: A Scoping Review
Source: Zoonoses Public Health. 2025 Jun 13;72(6):485–500. doi: 10.1111/zph.13230 (PMC12400019; doi:10.1111/zph.13230)
Supplement: Supplementary file 2 — Appendix S2. Database search strategies utilised for this scoping review. [file ZPH-72-485-s004.docx]

# Supplementary appendix 1: Database search strategies

## Embase

1. Exp foxes/ or (fox or foxes or vulpes* or vulpine or cerdocyon or lycalopex or otocyon or urocyon or dusicyon).mp
2. Exp Bacterial zoonoses/ or exp Gram positive bacteria/ or exp Gram negative bacteria/ or exp Gram positive infections/ or exp Gram negative infections/ or exp Bacterial toxins/ or (“bacterial zoonoses” or “gram positive and gram negative bacteria” or “gram positive bacteria” or “gram negative bacteria” or anthrax or anthrax* or anthracis or Bordetel* or botulism or brucell* or Burkholderi* or Buruli or campylobacter* or cholera or clostridium or corynebacteri* or coxiell* or cryptospor* or diphtheri* or “e.coli” or “escherichia coli” or erisypel* or escherichia or francisell* or glanders or helicobacter* or johne’s or johnes* or klebsiell* or leprosy or leptospir* or listeri* or melioid* or mrsa or “methicillin resistant staphyolococcus aureus” or mycobacteri* or paratuberculos* or pasteurell* or plague or pleuropneumonia or “q fever” or salmonell* or staphylococc* or streptococc* or tuberculo* or tularaemia or tularemia or weil* or yersini*).mp
3. Exp disease transmission/ or (transmission or spread or transmit* or reservoir* or epidemiolog* or host* or outbreak* or role* or sentinel or spillover or shed*).mp

1 AND 2 AND 3 = 619 results

## PubMed

1. Foxes[mh] OR fox[tw] OR foxes[tw] OR vulpine[tw] OR vulpes*[tw] OR cerdocyon[tw] OR lycalopex[tw] OR otocyon[tw] OR Urocyon[tw] OR dusicyon[tw]
2. Bacterial zoonoses[mh] OR Gram positive bacteria[mh] OR Gram negative bacteria[mh] OR Gram positive infections[mh] OR Gram negative infections[mh] OR Bacterial toxins[mh] OR “bacterial zoonoses”[tw] OR “gram positive and gram negative bacteria”[tw] OR “gram positive bacteria”[tw] OR “gram negative bacteria”[tw] OR anthrax[tw] OR anthrax*[tw] OR anthracis[tw] OR Bordetel*[tw] OR botulism[tw] OR brucell*[tw] OR Burkholderi*[tw] OR Buruli[tw] OR campylobacter*[tw] OR cholera[tw] OR clostridium[tw] OR corynebacteri*[tw] OR coxiell*[tw] OR cryptospor*[tw] OR diphtheri*[tw] OR “e.coli”[tw] OR “escherichia coli”[tw] OR erysipel*[tw] OR escherichia[tw] OR francisell*[tw] OR glanders[tw] OR helicobacter*[tw] OR johne’s[tw] OR johnes*[tw] OR klebsiell*[tw] OR leprosy[tw] OR leptospir*[tw] OR listeri*[tw] OR melioid*[tw] OR mrsa[tw] OR “methicillin resistant staphyolococcus aureus”[tw] OR mycobacteri*[tw] OR paratuberculos*[tw] OR pasteurell*[tw] OR plague[tw] OR pleuropneumonia[tw] OR “q fever”[tw] OR salmonell*[tw] OR staphylococc*[tw] OR streptococc*[tw] OR tuberculo*[tw] OR tularaemia[tw] OR tularemia[tw] OR weil*[tw] OR yersini*[tw]
3. Disease transmission, infectious[mh] OR transmission[tw] OR spread[tw] OR transmit*[tw] OR reservoir*[tw] OR epidemiolog*[tw] OR host*[tw] OR outbreak*[tw] OR role[tw] OR sentinel[tw] OR spillover[tw] OR shed*[tw]

1 AND 2 AND 3 = 567 results

## CAB Abstracts [EBSCO]

1. SU (fox OR foxes OR vulpine OR vulpes* OR cerdocyon OR lycalopex OR otocyon OR urocyon OR dusicyon) --> 9,752 results
2. SU (“bacterial zoonoses” or “gram positive and gram negative bacteria” or “gram positive bacteria” or “gram negative bacteria” or "bacterial toxins" or anthrax* or anthracis or Bordetel* or botulism or brucell* or Burkholderi* or Buruli or campylobacter* or cholera or clostridium or corynebacteri* or coxiell* or cryptospor* or diphtheri* or “e.coli” or “escherichia coli” or erysipel* or escherichia or francisell* or glanders or helicobacter* or johne’s or johnes* or klebsiell* or leprosy or leptospir* or listeri* or melioid* or mrsa or “methicillin resistant staphyolococcus aureus” or mycobacteri* or paratuberculos* or pasteurell* or plague or pleuropneumonia or “q fever” or salmonell* or staphylococc* or streptococc* or tuberculo* or tularaemia or tularemia or weil* or yersini*) --> 410,857 results
3. SU (transmission OR spread OR transmit* OR reservoir* OR epidemiolog* OR host* OR outbreak* OR role OR sentinel OR spillover OR shed*) --> 651,261 results

#1 AND #2 AND #3 = 246 results

## Google Scholar

fox AND (bacteria OR buruli) AND (transmission or spread or transmit* or reservoir* or epidemiolog* or host* or outbreak* or role* or sentinel or spillover or shed*)

Hand searched first 10 (of 265) pages, imported 5 refs

## Cochrane trials

**fox OR foxes OR vulpes** in Title Abstract Keyword AND **bacterial OR mycobacterial OR zoonoses** in Title Abstract Keyword - (Word variations have been searched)

10 results, 0 imported
