# Supplementary material for: The Role of Foxes in Transmitting Zoonotic Bacteria to Humans: A Scoping Review
Source: Zoonoses Public Health. 2025 Jun 13;72(6):485–500. doi: 10.1111/zph.13230 (PMC12400019; doi:10.1111/zph.13230)
Supplement: Supplementary file 3 — Appendix S3. Instructions for screeners. [file ZPH-72-485-s003.docx]

# Supplementary document 2. Screening instructions

## Title and abstract screening

Eligibility of studies was assessed based on titles and abstracts using the following questions:

1. Is the title/abstract in English? YES = [INCLUDE]; NO = [EXCLUDE]
2. Does the study concern transmission of or exposure to zoonotic bacteria in humans via foxes? YES = [INCLUDE]; NO = [EXCLUDE]; UNCLEAR = [INCLUDE]
3. Does the study concern exclusively blood- or vector-borne zoonotic bacteria? YES = [EXCLUDE]; NO = [INCLUDE]; UNCLEAR = [INCLUDE]
4. Is the study presenting primary research data? YES = [INCLUDE]; UNCLEAR = [INCLUDE]; NO = [EXCLUDE]

## Full text screening

Eligibility of full text articles was assessed via the following questions:

1. Is the full text available in English? YES = [INCLUDE]; NO = [EXCLUDE]
2. Does the study concern transmission of or exposure to zoonotic bacteria in humans via foxes? YES = [INCLUDE]; NO = [EXCLUDE]; UNCLEAR = [INCLUDE]
3. Does the study concern exclusively blood- or vector-borne zoonotic bacteria? YES = [EXCLUDE]; NO = [INCLUDE]; UNCLEAR = [INCLUDE]
4. Is the study presenting primary research data? YES = [INCLUDE]; UNCLEAR = [INCLUDE]; NO = [EXCLUDE]

Full text articles marked for exclusion were also assigned one of four reasons, in order of hierarchy:

1. No full text available
2. Article not in English
3. Not describing transmission or exposure to zoonotic bacteria of interest from foxes
4. Not presenting primary research data
