# Supplementary material for: The Role of Foxes in Transmitting Zoonotic Bacteria to Humans: A Scoping Review
Source: Zoonoses Public Health. 2025 Jun 13;72(6):485–500. doi: 10.1111/zph.13230 (PMC12400019; doi:10.1111/zph.13230)
Supplement: Supplementary file 4 — Appendix S4. Data extraction template. [file ZPH-72-485-s005.docx]

# Supplementary document 3. Data extraction template

| **General information** | |
| --- | --- |
| Characteristic | Options (if available) |
| Study ID |  |
| Author surname & publication year |  |
| Country in which study was conducted |  |
| Possible conflicts of interest for reviewers |  |
| Any other comments |  |
| **Study methodology** | |
| **Study overview** | |
| Study aim/objectives |  |
| Study design/methodology |  |
| Time period when study took place |  |
| Region/setting where study took place |  |
| Study setting(s) | - Urban - Semi-urban (eg suburbs) - Rural (eg small towns, farms, countrysides) - Other: |
| **Study population** | |
| Human population description (eg local residents, poultry farmers, fox hunters) |  |
| Eligibility criteria |  |
| Sampling strategy |  |
| **Bacteria of interest** | |
| Zoonotic bacteria described in the study (Latin name) |  |
| Disease described in the study (common name) |  |
| Features of infection/clinical presentation, if stated | - Host - Clinical signs - Comments |
| **Sampling and results** | |
| Sample type(s) collected |  |
| Sample testing method(s) |  |
| Hosts/environments sampled | - Humans - Foxes - Soil - Water - Other: |
| Other sample testing or methodology information or comments |  |
| Test results of interest (humans, foxes, soil/water) | - Sample type - No. tested - No. positive results - % positive results |
| **Fox data** | |
| Species of fox/es sampled, if applicable (common & scientific names) |  |
| Source/s of fox/es, if applicable | - Wild - Farmed - Laboratory - Other: |
| Route/s of infection in foxes (if known, or hypothesized) |  |
| Infection type in foxes (if known) | - Natural - Experimental - Other: |
| Fox bacterial shedding routes (if known) | - Oral fluids - Faeces - Sexual/reproductive fluids - Blood - Not known/stated - Other: |
| **Transmission / exposure from foxes to humans** | |
| Exposure type | - Confirmed transmission from fox - Suspected transmission/exposure from fox |
| Type of transmission/exposure from fox to human (if known, or hypothesized) | - Direct - Indirect - Not known |
| Route of transmission/exposure from a fox(es) to humans (if known, or hypothesized) |  |
| Strength of disease association with a fox(es) | - High - Medium - Low |
| Risk factors for transmission of zoonotic bacteria from foxes to humans (if known) |  |
| Any other comments |  |
